# *Acinetobacter baumannii* Resistance to Sulbactam/Durlobactam: A Systematic Review

**DOI:** 10.3390/antibiotics11121793

**Published:** 2022-12-10

**Authors:** Luigi Principe, Stefano Di Bella, Jacopo Conti, Mariagrazia Perilli, Alessandra Piccirilli, Cristina Mussini, Giuliana Decorti

**Affiliations:** 1 Clinical Pathology and Microbiology Unit, “San Giovanni di Dio” Hospital, 88900 Crotone, Italy; 2 Clinical University Department of Medical, Surgical and Health Sciences, University of Trieste, 34129 Trieste, Italy; 3 AOU Policlinico di Modena, Università degli Studi di Modena e Reggio Emilia, 41121 Modena, Italy; 4 Department of Biotechnological and Applied Clinical Sciences, University of L’Aquila, 67100 L’Aquila, Italy; 5 Institute for Maternal & Child Health (I.R.C.C.S) Burlo Garofolo, 34129 Trieste, Italy

**Keywords:** *Acinetobacter*, sulbactam/durlobactam, sulbactam-durlobactam, resistance, susceptibility, resistances, efficacy

## Abstract

Infections caused by carbapenem-resistant *Acinetobacter baumannii* (CRAB) have limited therapeutic options. Sulbactam-durlobactam is a combination of two βlactamase inhibitors with activity against CRAB under phase 3 clinical investigation. We performed a systematic review on in vitro studies reporting *A. baumannii* resistances against sulbactam/durlobactam. We considered “resistant” species to be those with MIC ≥ 8 mg/L. Ten studies were included in the review (9754 tested isolates). Overall, 2.3% of *A. baumannii* were resistant to sulbactam/durlobactam, and this percentage rose to 3.4% among CRAB subgroups and to 3.7% among colistin-resistant strains. Resistance was 100% among metallo β-lactamase-producing strains. Overall, in 12.5% of cases, sulbactam/durlobactam resistance was associated with the production of NDM-1, in 31.7% of cases with the substitutions in the PBP3 determinants, and in the remaining cases the resistance mechanism was unknown. In conclusion, *A. baumannii* resistance towards sulbactam/durlobactam is limited, except for MBL-producing strains.

## 1. Introduction

*Acinetobacter baumannii* infections are among the most difficult bacterial infections to manage. The difficulty largely arises from the antibiotic resistance profile of the bacterium, which is one of the most resistant microorganisms encountered in clinics. *Acinetobacter* displays multiple antibiotic resistance mechanisms (often coexisting) such as: enzymatic; non-enzymatic, involving efflux pumps and membrane permeability; and penicillin-binding proteins (PBPs) mutations [1,2].

Carbapenems have long been considered to be last-resort drugs for *Acinetobacter* infections, however during the last two decades we attended a global spread of carbapenem-resistant *Acinetobacter baumannii* (CRAB) strains that are at present in different countries around the world [3].

In the last few years, a number of new antibiotics against Gram-negative bacteria have been approved for human use. Most of them are β-lactam/β-lactamase inhibitor combinations (e.g., ceftazidime/avibactam, meropenem/vaborbactam, imipenem/relebactam) with no activity against CRAB [4]. Other new antibiotics are cefiderocol and eravacycline: these retain in vitro activity against CRAB, however cefiderocol experienced disappointing results in human studies [5] and the latter has virtually no human studies. In light of the above, CRAB remains the “big forgotten” in terms of therapeutic options.

Recently, the compound sulbactam/durlobactam entered phase 3 of a clinical trial. This is a new combination of two β-lactamase inhibitors. Sulbactam is a competitive, irreversible first generation β-lactamase inhibitor that has a direct-acting antibacterial activity against *A. baumannii*. Sulbactam, in high doses, saturates PBPs (PBP1 and PBP3) of *A. baumannii* isolates [6]. Durlobactam is a new member of the diazabicyclooctane class of β-lactamase inhibitors with broad spectrum activity against Ambler class A, C, and D serine β-lactamases [7]. Although sulbactam is an old drug (approved for medical use in 1986), it is still the preferred empirical agent according to the most recent guidelines [8].

However, there has been a steady decline in the susceptibility of *A. baumannii* to sulbactam [9,10] together with the resistance of *Acinetobacter* to carbapenems. Currently, less than 50% of CRAB are susceptible to sulbactam [11,12].

It is known that once *Acinetobacter* exhibits resistance to carbapenems, it usually has acquired several other antibiotic resistances, often including sulbactam [13]. To date, the resistance to sulbactam requires the addition of a second agent with a consequent potential increase of toxicity (e.g., nephrotoxicity with polymyxins) with no robust benefit on outcomes. In addition, it is known that the resistance to carbapenems confers a more than double mortality risk [14]. It is precisely with regard to CRAB isolates that the attention of researchers and clinicians is being directed, also in light of the ongoing phase 3 trial, on sulbactam/durlobactam.

We aim to provide an overview of the in vitro activity of sulbactam/durlobactam on CRAB isolates, also reporting resistance mechanisms and the geographical origin of the isolates, thus providing an “a priori” resistance estimate for clinicians facing CRAB infections.

## 2. Methods 

This systematic review was conducted in accordance with the PRISMA (Preferred Reporting Items for Systematic Reviews and Meta Analyses) guidelines. The review protocol was registered at the Prospero international prospective register of systematic reviews (ID 377051).

The systematic review was conducted sourcing the PubMed database. In order to include all the articles ever published with regard to this novel antimicrobial combination, two separate searches were performed using the generic items: “sulbactam/durlobactam” and “ETX2514”, the latter being the research name for durlobactam.

Only peer-reviewed articles written in English up to 25 September 2022 were assessed.

A reviewer screened all the titles and abstracts in order to determine the eligibility for full-text review. 

Inclusion criteria included full text availability, English language, and in vitro studies assessing *A. baumanii* resistance against sulbactam/durlobactam. Exclusion criteria were reviews, in vivo studies, PK/PD studies, case reports, expert opinions, and in vitro studies not concerning *A. baumannii*.

After initial screening, all eligible articles were assessed in full text, and data from each study was extracted. Data was then organized in a worksheet. The relevant data assessed was study author and publication year, region or country, collection period and type of sample (if available), pathogen, resistance determinants (where available), MIC range, number of isolates susceptible to sulbactam/durlobactam with MIC values ≤ 0.5 mg/L and MIC values ≤ 4 mg/L, methods used to evaluate interactions. 

No susceptibility breakpoint for sulbactam/durlobactam has been established yet, therefore each study had different MIC50 and MIC90: we considered susceptible species showing MIC ≤ 4 mg/L and highly susceptible species showing MIC ≤ 0.5 mg/L.

## 3. Results 

### 3.1. Literature Search 

**Figure 1:**
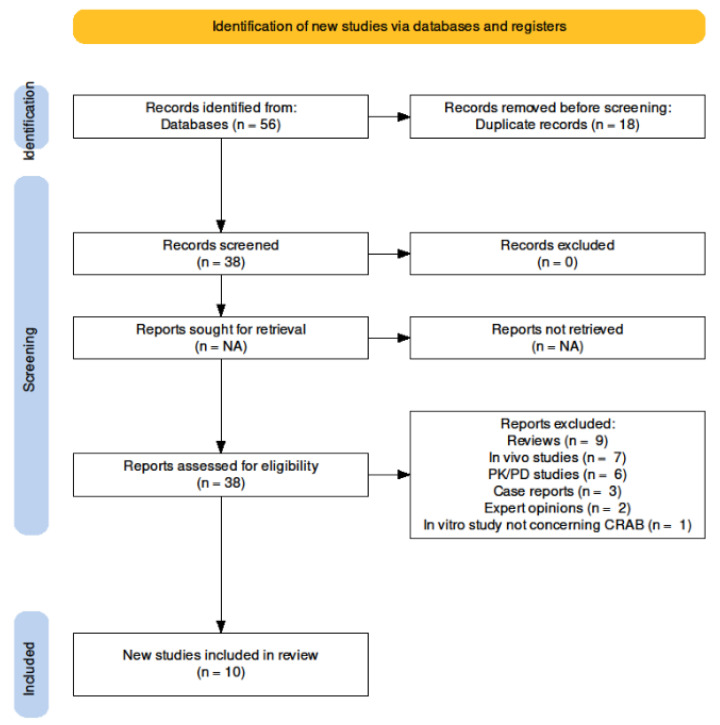
Search strategy.

The search method provided 56 references; after de-duplication, 38 studies were assessed: only in vitro studies concerning sulbactam/durlobactam activity against CRAB were deemed eligible, therefore 28 studies were eventually excluded due to the reasons listed in Figure 1, wherein the selection process is explained. Overall, 10 in vitro studies were included.

### 3.2. Microbiological Findings

The in vitro antimicrobial activity of the sulbactam/durlobactam combination was evaluated against a total of 9754 isolates belonging to the *A. baumannii* complex. All testings were performed by broth microdilution. The sulbactam/durlobactam combination showed in vitro antimicrobial activity against 9530 isolates (97.7%) (MIC ≤ 4 mg/L), while 224 isolates (2.3%) showed a resistant profile. In particular, sulbactam/durlobactam showed a high antimicrobial activity against 1209 isolates (12.4%), with MIC values ≤ 0.5 mg/L. MIC50 and MIC90 ranged from 0.25 to 4 mg/L and from 1 to 8 mg/L, respectively. MIC values of resistant strains ranged from 8 to >128 mg/L.

Among the 9754 *A. baumannii*, 5812 (59.6%) were carbapenem-resistant. Regarding CRAB, sulbactam/durlobactam was active (MIC ≤ 4 mg/L) against 5614 (96.6%) of them, while 198 CRAB (3.4%) displayed a resistant profile. 

When data were reported, CRAB isolates produced various oxacillinase (OXA)-type determinants, with OXA-23-type, OXA-58-type, OXA-24-type, OXA-40-type, OXA-66-type, OXA-72-type and OXA-237-type as the most represented determinants. 

Interestingly, 507 isolates (5.2% of total isolates) showed a pandrug-resistant (PDR) profile, being resistant to both carbapenems and colistin. Among them, sulbactam/durlobactam showed antimicrobial activity against 488 isolates (96.2%), while 19 (3.7%) were sulbactam/durlobactam-resistant. 

When reported, molecular data showed that sulbactam/durlobactam-resistant isolates were mostly associated with the production of New Delhi metallo-β-lactamase-1 (NDM-1) (n = 28, 12.5% of sulbactam/durlobactam-resistant isolates) or more often to substitutions in the PBP3 determinant (n = 71, 31.7% of sulbactam/durlobactam-resistant isolates). For the remaining sulbactam/durlobactam-resistant isolates (n = 125 of total resistant isolates, 55.8%) the resistance mechanism was not investigated. Overall, the presence of NDM-1 was associated to MIC values > 32 mg/L for sulbactam/durlobactam, while lower MIC values were associated with substitutions in the PBP3 determinant. Reported substitutions were A515V (n = 40), Q488K and Y258H (n = 11, co-produced), T526S (n = 12), T337I (n = 1), G523V (n = 1), K235N (n = 1), F548I (n = 1), V146I (n = 1), A578T (n = 1), A370Y (n = 1) N392T (n = 1), I517N (n = 1), V656L (n = 1). Interestingly, four sulbactam/durlobactam-resistant isolates presented a mutated adeJ gene (two of them also presented substitutions in the PBP3 determinant), with consequent alteration of the relative efflux system. Resistance to sulbactam/durlobactam seems to not be associated with particular sequence type (ST) or with specific OXA-type. The characteristics of in vitro studies are shown in Table 1. Table 2 shows the antimicrobial activity of sulbactam/durlobactam against different groups of *A. baumannii* isolates.

## 4. Discussion 

Currently, *Acinetobacter* is the most “armored” bacterium, with significant therapeutic difficulties. The most promising antibiotic, cefiderocol, did not give the expected clinical results against CRAB [5]. There are a lot of expectations for sulbactam/durlobactam, being under a phase 3 trial, in light of the promising in vitro data. 

Sulbactam/durlobactam demonstrated a good intrapulmonary penetration ratio for epithelial lining fluid (ELF) to total plasma concentrations of both agents in healthy subjects (38% for durlobactam and 50% for sulbactam, and even higher, 41 and 81%, respectively, if the unbound drug concentrations were considered), supporting the use of the combination in the treatment of pulmonary infections caused by CRAB [25]. According to data collected from our systematic review, sulbactam/durlobactam maintained in vitro activity against 98% of *A. baumannii* isolates (overall), dropping to 97% in the CRAB subgroup and to 96% in the colistin-resistant subgroup. Resistance to sulbactam/durlobactam was mainly associated with substitutions in the PBP3 determinant (32%), generally near its active serine site (S336), the sulbactam-binding site, and with production of MBLs (12%). The presence of MBL was associated with higher MIC values (>32 mg/L) compared to substitutions in the PBP3 determinant. At the present time, no specific ST, OXA-type or geographic area seems to be associated with the resistance. 

From a clinical point of view, when facing MBL-producing isolates of *A. baumannii*, the option of sulbactam/durlobactam is not appealing, in fact this drug appears inactive against 100% of these isolates. The last guidelines of the Infectious Diseases Society of America contemplate ampicillin/sulbactam use also when nonsusceptibility is demonstrated, given the potential for sulbactam to saturate altered PBP targets [8]. If this could be extended also to sulbactam/durlobactam remains to be demonstrated. In infections associated with colistin resistant or MBL-producing *A. baumannii* second choice options should be considered, such as minocycline, tigecycline, eravacycline and cefiderocol. The addition of fosfomycin to the regimen (only as partner drug) could also be considered in the light of recent favourable evidence, although numerically small [26,27]. All the above mentioned drugs would be better administered in combination given the weak evidence of clinical efficacy.

Further data are necessary to elucidate the resistance mechanisms to the sulbactam/durlobactam combination in *A. baumannii* (more than the 50% of resistance cases were not investigated), in order to acquire microbiological and clinical strategies to preserve the therapeutic option. In conclusion, *A. baumannii* resistance towards sulbactam/durlobactam is limited except for the MBL-producing strains. 

## Figures and Tables

**Table 1:** In vitro studies on sulbactam/durlobactam antimicrobial activity against *Acinetobacter* isolates.

Ref.	Region/Country or Type of Collection, Collection Period	Bacterial Species	Carbapenem- R (%)	SUL/DUR-R Determinants	MIC Range, MIC50, MIC90 (mg/L)	Highly Susceptible Isolates (%) (MIC ≤ 0.5)	Susceptible Isolates (%) (MIC ≤ 4)	SUL/DUR-R Isolates (%)	Colistin-R (%)	Notes
[15]	Italy Multicentric (6 centres) (2004–2021)	*A. baumannii* complex	141 (100%)	Substitutions in PBP3	0.06–>128 MIC50: 0.5 MIC90: 4	80/141 (57%)	130/141 (92%)	11 (7.8%)	55 (39%)	2 colistin-R isolates were also SUL/DUR-R. All SUL/DUR-R isolates had PBP3 substitutions
[16]	33 countries across the Asia/South Pacific region, Europe, Latin America, the Middle East, and North America (2016–2021)	*A. baumannii-calcoaceticus* complex: 80.2% *A. baumannii*, 12.7% *A. pittii*, 5.9% *A. nosocomialis*, 1.1% *A. calcoaceticus*	2488 (49.4%)	N/A	≤0.03–>64 MIC_50: 1 MIC_90: 2	N/A	4948/5032 (98.3%)	84 (1.7%) 79 *A. baumannii* 4 *A. pittii* 1 *A. nosocomialis*	204 (40.5%)	84 CRAB were SUL/DUR-R; 4 colistin-R isolates were R also to SUL/DUR
[17]	Worldwide (N/A)	*A. baumannii* complex	100 (100%)	Substitutions in PBP (PBP1a, PBP1b, PBP2, and PBP3), NDM	0.06–64	N/A	71/100 (71%)	29 (29%) 14 isolates with PBP3 substitutions 5 NDM-producing isolates	9 (9%)	5 colistin-R isolates were also R to SUL/DUR; 73 OXA-23, 10 OXA-72, 6 OXA-40, 5 OXA-58, 5 NDM, 1 OXA-24
[18]	Greece (2015)	*A. baumannii* complex	190 (100%)	Substitutions in PBP3, NDM	0.06–64 MIC50: 4 MIC90: 8	2/190 (1%)	167/190 (87.9%)	23 (12.1%)	61 (32.1%)	5 colistin-R isolates were also SUL/DUR-R; all R isolates harbored OXA-23 and OXA-66, with PBP3 substitutions; 1 NDM isolate
[19]	South America (N/A)	*A. baumannii* complex	112 (100%)	No resistant isolates	0.25–4 MIC50: 1 MIC90: 4	N/A	112/112 (100%)	0 (0%)	21 (18.7%)	34 OXA-23, 48 OXA 24/40, 10 OXA-143, 1 OXA-58, 17 OXA-23 + OXA-72
[20]	Global, 37 countries and six world regions (2012–2016)	*A. baumannii* complex	246 (100%)	NDM-1	0.25–128 MIC50: 0.25 MIC90: 1	63/246 (25.6%)	237/246 (96.3%)	9 (3.7%)	10 (4.1%)	Colistin-R isolates were all susceptible to SUL/DUR; 4 SUL/DUR-R isolates harbored NDM-1. For 5 SUL/DUR-R isolates resistance determinants not assessed
[21]	China 22 sites, IAI, LRTI, SSTI, UTI (2016–2018)	*A. baumannii* complex	831 (84.6%)	N/A	≤0.03–>64 MIC50: 1 MIC90: 2	N/A	961/982 (97.9%)	21 (21.4%)	10 (1%)	2 colistin-R isolates were also R to SUL/DUR
[22]	Global: 31 countries across Asia/South Pacific, Europe, Latin America, the Middle East and North America. BSI, IAI, LRTI, SSTI, UTI (2016 -2017)	*A. baumannii-calcoaceticus* complex: *A. baumannii* (82.5%) *A. pittii* (13.5%) *A. nosocomialis* (3.5%) *A. calcoaceticus* (0.6%)	930 (54%)	NDM-1, substitutions in PBP3 (but also in PBP1, PBP2, PBP6), efflux/porin variants	≤0.03–>64 MIC50: 1 MIC90: 2	723/1722 (42%)	1683/1722 (97.7%)	39 (2.3%)	81 (4.7%)	SUL/DUR-R isolates were carbapenem-R, 1 colistin-R isolate was also R to SUL/DUR; 11 harbored NDM-1, 21 had PBP3 substitutions, 16 had efflux/porin variants
[23]	United States (N/A)	*A. baumannii* complex	43 (43.9%)	b-lactamases, substitutions in PBP, and efflux pumps (mutations)	0.25–64 MIC50: 1 MIC90: 2	26/98 (26.5%)	94/98 (95.9%)	4 (4.1%)	N/A	All SUL/DUR-R isolates presented mutated adeJ efflux component, 2 of them also had PBP3 substitutions
[24]	Worldwide, 38 countries; IAI, UTI, SSTI, BSI, LRTI (2014)	*A. baumannii* complex	731 (64.6%)	NDM-1	≤0.06–32 MIC50: 1 MIC90: 4	315/1131 (27.9%)	1127/1131 (99.6%)	4 (0.4%)	56 (4.9%)	99.6% of SUL/DUR-R isolates were CRAB, none of them were colistin-R; 1 of them was NDM-1

BSI: bloodstream infections; IAI: intra-abdominal infections; LRTI: lower respiratory tract infections; R: resistant; SSTI: skin and soft tissue infections; SUL/DUR: sulbactam/durlobactam; UTI: urinary tract infections.

**Table 2:** Antimicrobial activity of sulbactam/durlobactam against *Acinetobacter* tested isolates.

Isolates Characteristics	Susceptible to SUL/DUR (%)	Resistant to SUL/DUR (%)
*A. baumannii* complex (n = 9754)	9530 (97.7%)	224 (2.3%)
CRAB (n = 5812)	5614 (96.6%)	198 (3.4%)
Colistin-resistant (n = 507)	488 (96.2%)	19 (3.7%)
NDM-1 producers (n = 28)	0 (0%)	28 (100%)

CRAB: carbapenem-resistant *Acinetobacter baumannii* complex; SUL/DUR: sulbactam/durlobactam.

## Data Availability

All data pooled and analyzed for this systematic review are included in the corresponding published articles, as reported in the main table.
